# A Preconception Paternal Fish Oil Diet Prevents Toxicant-Driven New Bronchopulmonary Dysplasia in Neonatal Mice

**DOI:** 10.3390/toxics10010007

**Published:** 2021-12-27

**Authors:** Jelonia T. Rumph, Kayla J. Rayford, Victoria R. Stephens, Sharareh Ameli, Pius N. Nde, Kevin G. Osteen, Kaylon L. Bruner-Tran

**Affiliations:** 1 Women’s Reproductive Health Research Center, Department of Obstetrics and Gynecology, Vanderbilt University School of Medicine, 1161 21st Ave S, MCN B-1100, Nashville, TN 37232, USA; jrumph19@email.mmc.edu (J.T.R.); victoria.r.stephens@vanderbilt.edu (V.R.S.); s.ameli@vanderbilt.edu (S.A.); Kevin.osteen@vanderbilt.edu (K.G.O.); 2 Department of Microbiology, Immunology and Physiology, Meharry Medical College, Nashville, TN 37208, USA; krayford@mmc.edu (K.J.R.); pnde@mmc.edu (P.N.N.); 3 Department of Pharmacology, Vanderbilt University, Nashville, TN 37208, USA; 4 Department of Pathology, Microbiology and Immunology, Vanderbilt University School of Medicine, Nashville, TN 37208, USA; 5 VA Tennessee Valley Healthcare System, Nashville, TN 37208, USA

**Keywords:** multigenerational, toxicants, bronchopulmonary dysplasia, therapeutics, lung development

## Abstract

New bronchopulmonary dysplasia is a developmental lung disease associated with placental dysfunction and impaired alveolarization. Risk factors for new BPD include prematurity, delayed postnatal growth, the dysregulation of epithelial-to-mesenchymal transition (EMT), and parental exposure to toxicants. Our group previously reported that a history of paternal toxicant exposure increased the risk of prematurity and low birth weight in offspring. A history of paternal toxicant exposure also increased the offspring’s risk of new BPD and disease severity was increased in offspring who additionally received a supplemental formula diet, which has also been linked to poor lung development. Risk factors associated with new BPD are well-defined, but it is unclear whether the disease can be prevented. Herein, we assessed whether a paternal fish oil diet could attenuate the development of new BPD in the offspring of toxicant exposed mice, with and without neonatal formula feeding. We investigated the impact of a paternal fish oil diet preconception because we previously reported that this intervention reduces the risk of TCDD associated placental dysfunction, prematurity, and low birth weight. We found that a paternal fish oil diet significantly reduced the risk of new BPD in neonatal mice with a history of paternal toxicant exposure regardless of neonatal diet. Furthermore, our evidence suggests that the protective effects of a paternal fish oil diet are mediated in part by the modulation of small molecules involved in EMT.

## 1. Introduction

The Developmental Origin of Health and Disease (DOHaD) concept was first proposed by Dr. David Barker who originally recognized the relationship between maternal malnutrition and the risk of metabolic syndrome in their adult children [1,2]. In recent years, the DOHaD concept has expanded and now recognizes a wide variety of factors that impact (positively or negatively) the fetal environment and, by extension, the child’s adult health [3,4,5]. Although originally focused on maternal factors relevant to the current study (diet, stress, environmental exposures), it now recognizes that the health of the paternal parent can also significantly impact the fetal environment and offspring health.

The father’s biological contribution to pregnancy is contained within his seminal fluid at the time of copulation. In addition to the spermatozoa, seminal fluid contains a variety of nutrients (fructose, citric acid), microbes, and proteolytic enzymes that are necessary for sperm survival and the successful fertilization of the oocyte [6,7,8]. Numerous studies have demonstrated that the quality of the seminal fluid can influence embryonic implantation, the microbial composition of the intrauterine environment, and placental function [9,10,11,12,13]. Importantly, the placenta is largely a paternally derived organ and is critical for pregnancy maintenance and fetal development [14]. For this reason, poor seminal fluid quality has the potential to not only negatively impact the development and function of the placenta but also to negatively impact fetal development and long-term child health.

Smoking and exposure to pollution are factors that reduce the quality of the seminal fluid and contribute to the DOHaD. Our laboratory previously reported that in utero exposure to 2,3,7,8-tetrachlorodibenzo-p-dioxin (TCDD)—a byproduct of smoke and a contributor to pollution—subsequently reduced the number and quality of sperm in male mice of reproductive age [10]. Furthermore, the offspring of these mice were susceptible to premature birth and intrauterine growth restriction (IUGR) [15,16]. We later demonstrated that these complications were associated with TCDD associated placental dysfunction that is characterized by alterations in the size of the placenta as well as the expression of placental progesterone receptor and toll-like receptor-4, which each play integral roles in placental function and pregnancy outcomes [10].

Paternal diet has also been shown to influence the DOHaD by modulating the quality of seminal fluid [17]. Our group and others have reported that a paternal fish oil diet improves the quality of seminal fluid. We found that providing a preconception supplemental fish oil diet to male mice that were exposed to TCDD in utero markedly improved both sperm density and motility and was associated with a significant increase in fertility. The improvement in sperm quality also translated to enhanced placental function, marked by a reduced expression of toll-like receptor 4 and increased expression of the progesterone receptor. Intervening with a paternal fish oil diet also reduced the risk of premature birth and eliminated the incidence of offspring IUGR [18].

In human neonates, premature birth and IUGR are each associated with an increased risk of developing new BPD, a developmental lung disease that is characterized by impaired alveolarization [19,20]. Several studies have suggested that placental dysfunction precedes (and contributes to) the development of new BPD [21,22,23,24]. The onset and severity of developmental lung diseases, such as new BPD, have also been linked to exposure to pollution in humans and experimental animal models [25,26,27,28,29]. Our laboratory found that the offspring of male mice that were exposed to TCDD in utero were also susceptible to new BPD [29]. Herein, we examined the efficacy of a paternal fish oil diet to prevent this potentially fatal neonatal disease. Our study revealed markedly improved lung development and reduced incidence of new BPD in offspring sired by a father with a history of TCDD exposure that was also provided a fish oil diet preconception.

Finally, exposure to components of fish oil and TCDD have been found to influence proteins that help to regulate epithelial-to-mesenchymal transition (EMT) within the lung; a process that is linked to the development of new BPD [30,31,32,33]. Therefore, we investigated whether a paternal fish oil diet attenuated the development of new BPD by modulating the small molecules involved in EMT. We also observed how neonatal diet impacted these parameters because maternal milk is associated with better infant health outcomes than formula feeding, which has been linked to poor postnatal lung development [34,35]. We found that the reduced risk of new BPD in the offspring of fish oil-supplemented males was associated with significantly reduced beta-catenin gene expression—a molecule involved in EMT and the development of new BPD [36,37,38,39].

## 2. Materials and Methods

### 2.1. Animals

Adult (10–12 weeks) and neonatal C57BL/6 mice were used in this study. Adult mice were obtained from Envigo (Indianapolis, IN, USA) or born in-house. All neonatal mice were born in-house. The animals were housed in the Barrier Animal Care Facility at the Vanderbilt University Medical Center, which is free from common mouse pathogens. Adult mice were provided food and water ad libitum. Animal room temperatures were maintained between 22–24 °C with a relative humidity of 40–50% on a 12-h light–dark schedule. Experiments described in this study were approved by Vanderbilt University’s Institutional Animal Care and Use Committee (IACUC) as per the Animal Welfare Act protocol #M2000098. Approval date: 1 December 2019.

### 2.2. Chemicals

TCDD (99% in nonane #ED-908) was purchased from Cambridge Isotope Laboratories (Andover, MA, USA). Esbilac Puppy Milk Replacer Powder was purchased from Pet-Ag, Inc (Hampshire, IL, USA). All other chemicals were obtained from Sigma-Aldrich (St. Louis, MO, USA) unless otherwise stated.

### 2.3. TCDD Exposure and Mating Scheme

Virgin 10- to 12-week-old C57BL/6 females were mated with intact males of a similar age. The females were weighed daily and monitored for the presence of a vaginal semen plug, denoting that copulation had occurred. The morning a vaginal plug was identified, the dam was considered pregnant (denoted as embryonic day 10^0.5^ and moved to a new cage. Following the confirmation of pregnancy, dams were exposed to TCDD (10 µg/kg) in corn oil or corn oil vehicle alone by gavage on 10^15.5^ at 1100 h CST. The dams provided with vehicle only were used as unexposed controls while dams receiving TCDD were designated F0 mice (the founding generation).

Although the selected dose of TCDD is higher than typical human exposures, this dose reflects the more rapid clearance of this toxicant in mice compared to humans. This dose is well below the LD50 for adult C57BL/6 mice (230 µg/kg) [40] and is not overtly teratogenic or abortogenic. In our hands, parturition typically occurred on E20 for both control and F0 pregnancies. Finally, since the half-life of TCDD is 11 days in C57BL/6 mice, the offspring of the F0 dams (F1 pups) were directly exposed to TCDD in utero and during lactation [40]. Germ cells present within F1 fetuses were also directly exposed to TCDD, and these cells had the potential to become the F2 generation.

### 2.4. Diet and Mating Scheme for the F1 Generation

Purina Mills (TestDiet division) provided the 5% Menhaden fish oil diet, which also contained 1.5% corn oil to prevent the depletion of omega-6 fatty acids. Menhaden fish oil, (OmegaProtein, Houston, TX, USA) has an established fatty acid profile (~40% omega-3 fatty acids) and was processed by the manufacturer to remove dioxins and polychlorinated biphenyls. The fish oil diet is a modification of Purina’s low phytoestrogen rodent chow 5VR5, which was used as the control (standard) diet. Protein, total fat, and energy content was the same for each diet. The fish oil diet was maintained in vacuum-sealed bags at −20 °C until use and once provided to mice, it was replaced every 3 days.

After weaning, F1 and control males were maintained on a standard or fish oil diet for at least 7 weeks (one full cycle of spermatogenesis) and mated at 10–12 weeks of age with age-matched unexposed C57BL/6 females. Once a vaginal semen plug was identified, dams were singly housed until parturition. Offspring sired by control males were denoted as CT whereas the offspring of F1 males were denoted F2TCDD.

### 2.5. Formula Feeding

Beginning on postnatal day 7 (PND7), CT and F2TCDD pups were sexed, weighed, and randomized to a strict maternal milk diet or a supplemental formula diet. Pups were bottle-fed 30 µL of formula three times a day over the course of four days using a small nipple attached to a 1ml syringe (Miracle Nipple Mini for Pets and Wildlife). Each 30 µL dose was provided in two aliquots of 15 µL, each 10 min apart. All pups remained with dams for the duration of the study and were allowed to nurse ad libitum. Pups were weighed daily and monitored for macroscopic signs of new BPD (e.g., labored breathing and difficulty feeding) from PND 7–10.

### 2.6. Euthanasia and Collection of Tissue

To assess the development of new BPD, we performed necropsies on PND 11 as our previous studies revealed that the disease was prevalent by this time in F2TCDD pups [29]. On PND 11 at 1100 h local time, CT and F2TCDD pups were weighed and observed for external signs of new BPD (labored breathing and/or delayed growth), then euthanized by decapitation performed under deep anesthesia as per the AAALAC guidelines. Following euthanasia, the peritoneal cavity and rib cage were opened, and the lungs and trachea were isolated and weighed. The lungs were perfused and inflated using an intratracheal injection of 1× phosphate buffered saline (PBS) as previously described [41,42]. A 1mL syringe filled with PBS was inserted into the trachea and used to inflate the left lobe of the lung. A small string was tied around the right lobe to prevent inflation. The non-inflated lobe was stored at −80 °C for qPCR and immunoblot analyses and the inflated lobe was fixed in 10% buffered formalin. The fixed tissues were processed and paraffin-embedded, and slides containing 4 µm sections were prepared by Vanderbilt University’s Translational Pathology Shared Resource Center (TPSR).

### 2.7. Hematoxylin and Eosin (H and E) Staining

Formalin-fixed, paraffin-embedded tissue sections were mounted on slides, then deparaffinized in xylene and rehydrated in ethanol. Slides were then incubated in Hematoxylin, washed, and incubated in Eosin. The slides were dehydrated in ethanol and xylene, then coverslipped using Cytoseal XYL (Thermo Scientific, Waltham, MA, USA; Cat# 8312-4).

### 2.8. Alveolus Diameter and Radial Alveoli Count

The measurements of alveoli diameter and radial alveolar count were conducted as previously described by others [43,44,45]. The linear ruler on Image J was used to assess the horizontal length of the individual alveoli that developed in the alveolar space of each group. Measurements were taken using the horizontal length of the alveoli to account for irregularities in alveolar shape/branching. H and E-stained slides from at least six pups from six separate litters were used to determine the average alveolus diameter for each group. At least 10 measurements were taken per 100× image to obtain the average alveolus diameter per pup. The manual measurements of the pulmonary alveolar space were confirmed using the mean linear intercept, a semi-quantitative method for lung morphology that has been used previously [46]. For the radial alveolar count, a line was drawn from the surface epithelium to the nearest bronchiole. Alveoli that intersected the line were counted and the average was taken for each group in the same manner as described above.

### 2.9. Histological Determination of BPD

We developed an unbiased lung scoring system to identify mice that did or did not have new BPD. The diagnosis of new BPD was determined using a lung injury scale that accounted for alveoli diameter, radial alveolar count, and the presence of red blood cells (RBCs) in the lung. We measured these parameters because reduced alveolus diameter and radial alveolar count are established markers for reduced lung alveolarization and impaired lung function [47]. Furthermore, the altered distribution of RBCs in the lung is related to respiratory distress and hypertension, which are associated with new BPD [48,49,50,51]. We carefully assessed multiple sections from 10 non-littermate CT pups to determine the normal range of these parameters.

Healthy pups had an average alveolus diameter of 25 ± 5 µM and an average radial alveolar count of 5–7. Healthy pups also exhibited little to no RBCs infiltrating the alveolar space. A lung injury score of 1–4 was indicative of a healthy lung, marked by the formation of distinct alveoli. Pups were considered to have mild BPD when they exhibited an average alveolus diameter of 20 ± 4 µM, an average radial alveolar count of 3–4, and the presence of RBCs in 20–50% of the alveolar space. Pups with mild BPD received a lung injury score of 5–7. Pups diagnosed with severe BPD exhibited an average alveolus diameter of 16 µM or less, an average radial alveolar count of 3 or less, and the presence of RBCs in more than 50% of the alveolar space. Pups with severe BPD were given a lung injury score of 8 or 9. To date, pups have not exhibited overlapping pulmonary phenotypes.

### 2.10. qRT-PCR

Lung tissue was lysed using the Trizol reagent (Invitrogen, Carlsbad, CA, USA) and the total RNA was purified from tissue lysates using the RNeasy Mini Kit (Qiagen, Valencia, CA, USA). An iScript cDNA synthesis kit was used to generate cDNA from 1 µg of the total RNA (Bio-Rad) using random decamer primers. The same thermal cycling program was applied to all primers: 95 °C for 30 s, 40 cycles of 95 °C for 5 s, and 60 °C for 5 s using a Bio-Rad CFX96 real-time thermocycler. The melting curve was analyzed to confirm product purity. All reactions were performed in triplicate. A *Ribosomal 18s* transcript was used as a housekeeping gene to normalize the transcript levels of E-cadherin and β-catenin for all samples. The results were evaluated using the delta-delta Ct method as previously described [15,18]

### 2.11. Immunoblot Assays

Lung tissue was homogenized in a RIPA buffer (Life Technologies, cat# R0278; CA, USA), containing a protease inhibitor cocktail set III at 1:100 (Calbiochem, Gibbstown, NJ, USA) and phosphatase inhibitor cocktails 2 and 3 at 1:100 each (Sigma Aldrich, St. Louis, MO, USA), and using a Tissue Tearor (United Lab Plastics, St. Louis, MO, USA). Lysates (20 μg/well) were separated by SDS-PAGE using 4–15% gradient polyacrylamide gels and transferred onto nitrocellulose membranes (Life Technologies, Carlsbad, CA, USA). The membranes were blocked in Intercept TBS Blocking Buffer (LI-COR Biosciences, Lincoln, NE, USA) and then incubated with an appropriate primary antibody diluted at 1:1000 in the blocking buffer at 4 °C overnight on a shaker: rabbit anti-beta-Catenin antibody (Cell Signaling Technology, Danvers, MA, USA; Cat# 8480); mouse anti-e-cadherin (Cell Signaling Technology, Danvers, MA, USA; Cat# 3195). Blots were incubated in a mouse anti-beta-actin monoclonal antibody (Sigma Aldrich, MO, USA; Cat# C7207) for 1 h at room temperature. The blots were washed and incubated with the IRDye 680RD Donkey anti-Mouse or IRDye 800 CW Goat anti-Rabbit (Licor, Lincoln, NE; cat# 926-68072 and 925-3221) secondary antibody in the blocking buffer containing 0.01% Tween 20 for 1 h at room temperature. The blots were washed with 1X TBS 0.01% Tween 20 and the bound antibody bands were scanned using the infrared fluorescence detection Odyssey Imaging System (LI-COR Biosciences). The housekeeping beta-actin signal was used for the normalization of the data. Each experiment was conducted in biological triplicates and the quantitation of band intensity was performed by densitometry using Image J.

### 2.12. Statistical Analysis

The alveolar measurements, qRT-PCR, and immunoblot data were analyzed using GraphPad Prism’s one-way ANOVA and the Tukey post-hoc test. For all experiments, six non-littermates were used to obtain the average for each group. The presented images are representative of each group. The data are represented as the mean ± standard deviation. *p* < 0.05 were considered significant. Significance was determined by comparing each treatment group to maternal milk-fed CT pups. All experiments were repeated twice using different non-littermates. In each group, approximately half of the pups were male and the other half were female. The majority of the pups were male in groups with uneven samples sizes.

## 3. Results

### 3.1. A Paternal Fish Oil Diet Preconception Improves Postnatal Growth in Pups with a History of TCDD Exposure

**Figure 1:**
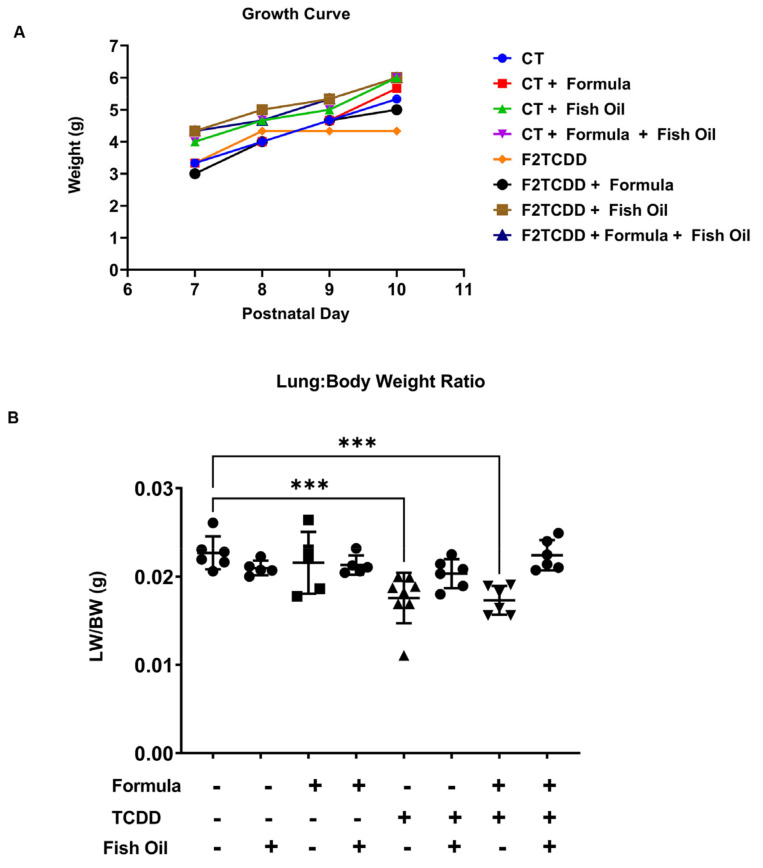
A dietary fish oil intervention improves postnatal development in pups: CT and F2TCDD pup body weight was monitored from postnatal day 7–10 (**A**), lung hypoplasia was determined by measuring lung-to-body weight ratios in CT and F2TCDD pups (**B**). Growth curve data represents the mean value of 4–5 non-littermate pups. Lung-to-body weight ratio data represents individual values of 4–5 non-littermate pups. Standard deviation is shown. *** *p* ≤ 0.001.

Infants diagnosed with new bronchopulmonary dysplasia (BPD) typically exhibit low birth weight, delayed postnatal growth, and lung hypoplasia [52,53,54]. To determine the efficacy of a preconception paternal fish oil diet intervention in eliminating factors associated with new BPD, we monitored pup growth from postnatal day (PND) 7–10 and assessed lung-to-body weight ratios on PND 11. Control (CT) pups had an average body weight of 3.3 g by PND 7, which increased to an average of 5.3 g by PND 10. Seven-day-old pups sired by fathers exposed to 2,3,7,8-tetrachlorodibenzo-p-dioxin in utero (F2TCDD pups) who were maintained on a standard diet preconception had a similar average body weight to CT pups. By PND 7, F2TCDD pups on a maternal milk diet had an average body weight of 3.3 g, which was reduced by 0.3 g in formula-supplemented pups. The average body weight of F2TCDD pups receiving maternal milk plateaued at 4 g by PND 8, but the average body weight of pups who received supplemental formula gradually increased to 5 g by PND 10. Regardless of their postnatal diet, F2TCDD pups sired by a father on a fish oil diet had an average body weight of 4.5 g by PND 7, which gradually increased to 6 g by PND 10. However, these trends in postnatal growth did not reach statistical significance (Figure 1A), as demonstrated in Table S1.

Lung hypoplasia manifests as a lung-to-body weight ratio of 0.0115, or 1.15%, or less [55,56]. On PND 11, CT pups displayed an average lung-to-body weight ratio of 0.022 g, or 2.2%. F2TCDD pups sired by a standard diet father exhibited an average lung-to-body weight ratio of 0.018, or 1.8%, when they received a strict maternal milk diet and 0.017, or 1.7%, when they received supplemental formula. F2TCDD pups were not diagnosed with lung hypoplasia, but they displayed a significant reduction in lung-to-body weight ratios compared to CT pups (*p* < 0.0005). Intervening with a paternal fish oil diet preconception normalized lung-to-body weight ratios to 0.020, or 2%, in maternal milk-fed and 0.021, or 2.1%, in formula-supplemented F2TCDD pups (Figure 1B). These results confirm our previous finding that a paternal fish oil diet intervention influences the offspring’s postnatal development [18]. Herein, we demonstrated that a paternal fish oil diet intervention improved postnatal growth rates and lung-to-body weight ratios in pups with a history of paternal TCDD exposure, regardless of their postnatal diet.

### 3.2. A Paternal Fish Oil Diet Mitigates Delayed Lung Development in Pups with a History of TCDD Exposure

**Figure 2:**
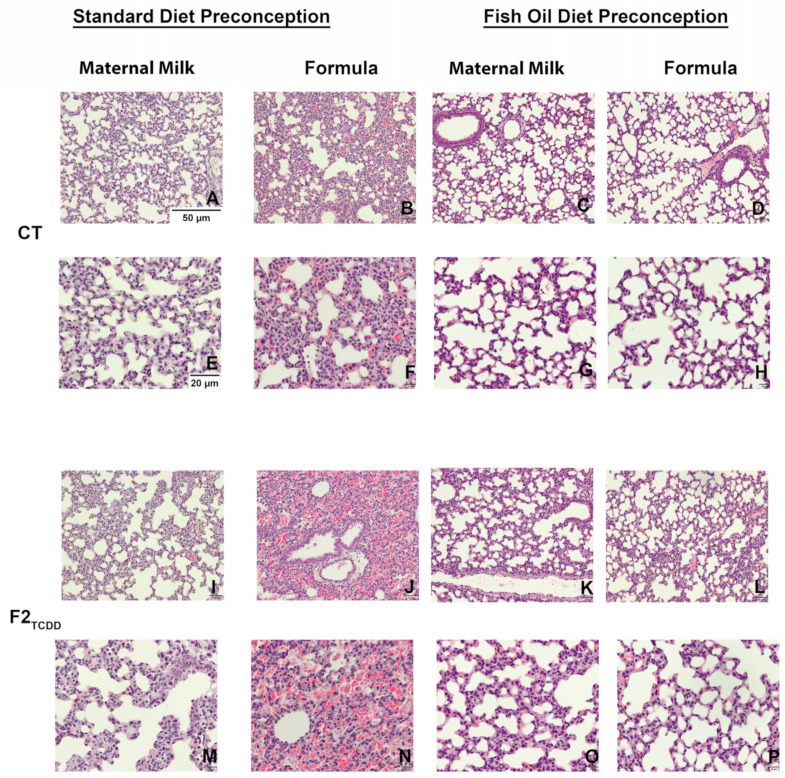
A paternal dietary fish oil intervention improves lung development in pups: Representative images of Hematoxylin- and Eosin-stained perfused lung tissue of PND11 CT pups sired by a standard or fish oil diet father following a maternal milk diet or supplemental formula at a magnification of 100× (**A**–**D**) and 400× (**E**–**H**); F2TCDD pups sired by a standard or fish oil diet father following a maternal milk diet or formula supplementation at a magnification of 100× (**I**–**L**) and 400× (**M**–**P**).

To confirm that a paternal fish oil diet preconception improves lung development in pups, we observed pulmonary histology using hematoxylin- and eosin-stained (H and E) lung slides. CT pups exhibited normal lung development and alveolarization, marked by the distinct formation of alveoli (Figure 2A,E). Supplementing the CT pup diet with formula led to a visible reduction in alveolar space and the thickening of alveolar walls (Figure 2B,F). CT pups sired by a father who received a fish oil diet preconception exhibited improved lung alveolarization, regardless of their postnatal diet (Figure 2C,D,G,H).

Lung H and Es confirmed that F2TCDD pups sired by fathers on a standard diet exhibited an impaired development of distinct alveoli and thickening of alveolar walls (Figure 2I,M). When provided supplemental formula, many F2TCDD pups experienced interalveolar red blood cell (RBC) infiltration (Figure 2J,N). A paternal fish oil diet preconception improved lung alveolarization and reduced the risk of interalveolar RBC infiltration in F2TCDD pups, regardless of postnatal diet (Figure 2K,L,O,P).

**Figure 3:**
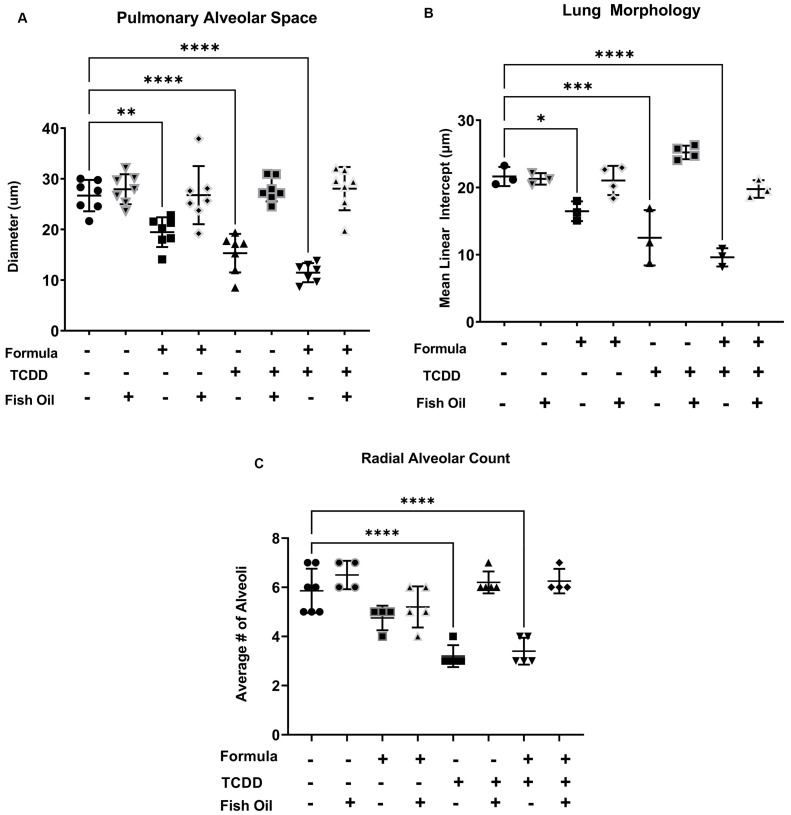
A paternal dietary fish oil intervention improves the alveolarization of pup lungs: Pulmonary alveolar space (**A**), mean linear intercept (**B**), and radial alveolar count (**C**) of CT and F2TCDD pups ± a fish oil intervention and/or supplemental formula was measured on PND11. Groups used for manual determination of pulmonary alveolar space and radial alveolar count contained 6–10 non-littermates. Data points represent the mean values from individual pups. Standard deviation is shown. Groups used for automated mean linear intercept contained 3–4 non-littermates.* *p* ≤ 0.05;** *p* ≤ 0.01;*** *p* ≤ 0.001;**** *p* ≤ 0.0001.

H- and E-stained slides were used to assess lung morphology by manually measuring the pulmonary space and radial alveoli count as previously described [43,45]. The pulmonary alveolar space measurements were verified using mean line intercept (MLI), an automated semi-quantitative lung morphology analysis [46]. CT pups sired by a father on a standard diet preconception had an average alveolar diameter of 25 ± 5 µm. Supplementing CT pups with formula led to a significant reduction in the average alveolar diameter (*p* = 0.0027). A paternal fish oil diet intervention normalized the average alveolar diameter of formula-supplemented CT pups. F2TCDD pups sired by a standard diet father also exhibited a significant reduction in the diameter of their alveolar space, regardless of postnatal diet (*p* < 0.0001). Intervening with a paternal fish oil diet in F2TCDD pups normalized their alveolar diameter to that of CT pups (Figure 3A).

We confirmed that formula-fed CT pups sired by a standard diet father also exhibited a significant decrease in lung MLI (*p* < 0.0228). F2TCDD pups also exhibited a significant reduction in lung MLI, independent of postnatal diet (*p* = 0.0001) (Figure 3B). All CT pups exhibited an average number of alveoli of 6 ± 1, independent of their postnatal diet. F2TCDD pups sired by a father on a standard diet displayed a significant reduction in their average number of alveoli compared to CT pups (*p* < 0.0001). Intervening with a paternal fish oil diet preconception in F2TCDD pups normalized their average number of alveoli to that of CT pups (Figure 3C). Our results confirm formula-fed CT pups and formula-fed/maternal milk-fed F2TCDD pups exhibit poor postnatal lung development, marked by a reduction in alveolar diameter, lung MLI, and radial alveolar count. We also confirmed the hypothesis that intervening with a paternal fish oil diet preconception in these groups improves lung development by increasing the average diameter of alveolar space, lung MLI, and number of alveoli in the offspring.

### 3.3. A Paternal Fish Oil Diet Reduces the Incidence of New BPD in Pups with a History of TCDD Exposure

**Figure 4:**
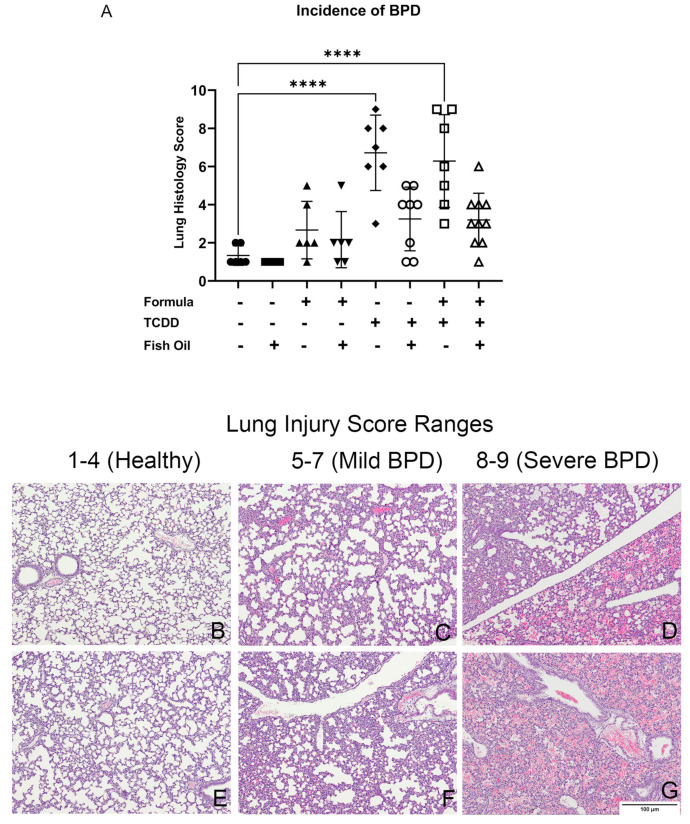
A dietary fish oil intervention reduces the incidence of BPD in TCDD-exposed pups: Incidence of BPD was determined in CT and F2TCDD pups ± a fish oil intervention and/or formula supplementation (**A**), the incidence of BPD was determined using a novel scale based on lung histology (**B**–**G**). Data points represent the individual lung injury scores of 6–10 non-littermates from each group. Standard deviation is shown. **** *p* ≤ 0.0001.

To determine the incidence of new BPD among pups, we used a scoring system based on relevant histologic markers, as detailed in the methods and demonstrated in Figure 4B–G. Regardless of their postnatal diet, F2TCDD pups sired by a father receiving a standard diet preconception displayed a significant increase in the incidence of new BPD compared to all CT pups (*p* < 0.0001) (Figure 4A). This translated to 85% of maternal milk-fed and 71% of formula-supplemented F2TCDD pups developing new BPD. Intervening with a paternal fish oil diet preconception reduced the incidence of new BPD to 25% in maternal milk-fed and 10% in formula-supplemented F2TCDD pups (Table 1). These findings support our hypothesis that a paternal fish oil diet preconception mitigates the development of new BPD in pups with a history of paternal TCDD exposure.

### 3.4. Diet and History of TCDD Exposure Influences Pup Pulmonary Beta-Catenin and E-Cadherin Expression

The small molecules beta-catenin and E-cadherin play integral roles in lung development. These proteins are involved in epithelial-to-mesenchymal transition (EMT)—a process that is dysregulated during the development and progression of new BPD [30]. Since EMT contributes to normal lung development and the development of lung diseases, we aimed to determine whether a paternal fish oil diet reduced the incidence of new BPD in offspring by modulating the small molecules associated with EMT. We examined pup beta-catenin and E-cadherin expression to observe whether a paternal fish oil diet intervention improves new BPD outcomes by influencing these small molecules.

**Figure 5:**
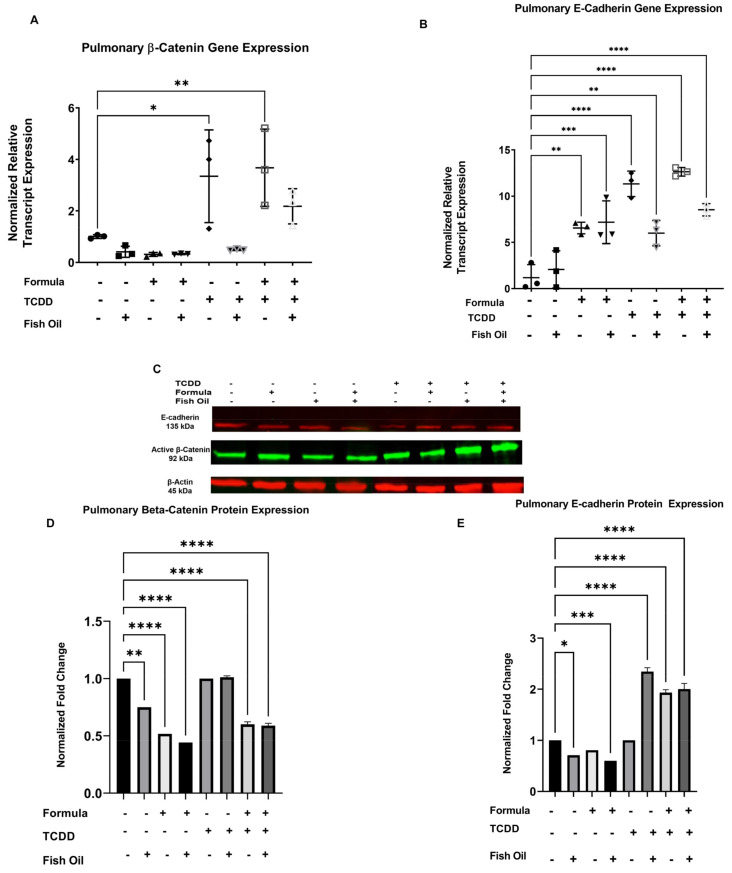
Diet and history of TCDD exposure influence beta-catenin and E-cadherin expression: RNA isolated from the lungs of PND 11 CT and F2TCDD pups ± a fish oil intervention and/or supplemental formula was used to quantify the expression of beta-catenin and E-cadherin gene expression (**A**,**B**), beta-catenin and E-cadherin protein expression were measured through immunoblotting (**C**), and quantified using densitometry (**D**,**E**). * *p* ≤ 0.05; ** *p* ≤ 0.01; *** *p* ≤ 0.001; **** *p* ≤ 0.0001.

CT pups displayed no differences in their expression of beta-catenin at the transcript level, regardless of paternal and postnatal diet. F2TCDD pups on a maternal milk diet (*p* = 0.0234) and supplemental formula diet (*p* = 0.0091) displayed a significant increase in beta-catenin gene expression when their father received a standard diet preconception. Intervening with a paternal fish oil diet preconception reduced beta-catenin gene expression in F2TCDD pups, regardless of postnatal diet (Figure 5A).

Significantly, compared to CT pups sired by a standard diet father and only provided maternal milk, E-cadherin gene expression was increased in CT pups receiving supplemental formula who were sired by fathers on a standard diet preconception (*p* = 0.0017). A paternal fish oil diet further increased E-cadherin gene expression in formula-supplemented CT pups (*p* = 0.0006). F2TCDD pups sired by standard diet fathers exhibited significant increases in E-cadherin gene expression, independent of postnatal diet (*p* < 0.0001). A paternal fish oil diet intervention in maternal milk-fed (*p* = 0.0044) and formula-supplemented F2TCDD pups (*p* < 0.0001) also significantly increased E-cadherin gene expression (Figure 5B).

We also quantified the protein expression of pulmonary beta-catenin and E-cadherin in pups through immunoblotting (Figure 5C and Figure S1). A paternal fish oil diet intervention significantly decreased beta-catenin protein expression in CT pups (*p* = 0.0020). Beta-catenin protein expression was further reduced in CT pups receiving supplemental formula, regardless of paternal diet preconception (*p* < 0.0001). Independent of paternal diet preconception, beta-catenin protein expression in F2TCDD pups was similar to maternal milk-fed CT pups sired by standard diet fathers. The formula supplementation of F2TCDD pups significantly reduced beta-catenin protein expression, regardless of paternal diet (*p* < 0.0001) (Figure 5D).

Significantly, CT pups sired by a father on a fish oil diet preconception exhibited a reduction in E-cadherin protein expression following a strict maternal milk diet (*p* = 0.0108) and a supplemental formula diet (*p* < 0.0001). F2TCDD pups sired by a standard diet father expressed similar levels of E-cadherin protein expression to maternal milk-fed CT pups sired by a standard diet father. Regardless of offspring postnatal diet, a paternal fish oil diet intervention in F2TCDD pups led to increased E-cadherin protein expression (*p* < 0.0001). The formula supplementation of F2TCDD pups sired by standard diet fathers also led to a significant increase in E-cadherin protein expression (*p* < 0.0001) (Figure 5E).

## 4. Discussion

We previously reported that a paternal history of TCDD exposure increased the offspring’s risk of premature birth and IUGR due to impaired placental function [18]. A paternal history of TCDD exposure also increased the offspring’s risk of new BPD, a disease previously associated with prematurity, IUGR, and placental dysfunction [19,21,22,57]. The severity of new BPD worsened in neonatal mice that received supplemental formula, although this dietary intervention is commonly provided to premature human infants to enhance their nutritional intake and promote lung development [29,58,59]. Intervening with a paternal fish oil diet preconception, following his own in utero TCDD exposure, reduced the risk of premature birth and IUGR in offspring in association with improved placental function [18]. Herein, we investigated the efficacy of a paternal fish oil diet preconception in preventing the development of new BPD in offspring with a history of paternal TCDD exposure. We also observed whether the protection offered by a paternal fish oil diet persisted independently of the offspring’s postnatal diet (maternal milk vs. supplemental formula).

Herein we confirmed that a paternal history of TCDD exposure impaired postnatal growth in offspring from PND 7 to 10. A paternal fish oil diet preconception improved offspring growth independent of postnatal diet; however, this trend did not reach significance (Figure 1A and Table S1). As demonstrated by Figure 1B, a history of paternal TCDD exposure also impaired lung development, denoted by an increased risk of lung hypoplasia, which is associated with poor lung health outcomes [60]. Intervening with a paternal fish oil diet preconception in males with a history of in utero TCDD exposure reduced the offspring’s risk of lung hypoplasia, independent of postnatal diet.

We also observed the lung histology of pups from each group and found that CT pups exhibited normal alveolarization when they received a postnatal maternal milk diet (Figure 2A,E). Our results confirmed that postnatal formula supplementation may be associated with impaired lung development [34,61], marked by the thickening of the alveolar walls in CT pups who received postnatal formula supplementation (Figure 2B,F). A paternal fish oil diet preconception also improved lung histology in CT pups provided with supplemental formula. The paternal history of TCDD exposure preceding a standard diet preconception impaired lung development in pups who received a maternal milk diet (Figure 2I,M) and a supplemental formula diet (Figure 2J,N). Additionally, formula supplementation led to interalveolar RBC infiltration—a sign of pulmonary hypertension, which is associated with the development of new BPD in human neonates [62,63]. Intervening with a paternal fish oil diet preconception in males with a history of TCDD exposure improved lung development in their offspring, independent of postnatal diet (Figure 2K–P). This intervention also reduced the risk of interalveolar RBC infiltration in formula-supplemented F2TCDD pups.

We used the previously described methods to assess pulmonary alveolar space, lung MLI, and radial alveolar count, which are altered in developmental lung diseases [64]. We confirmed that formula supplementation has a negative effect on the diameter of pulmonary alveolar space, as it was significantly reduced in CT pups who received supplemental formula (Figure 3A). We also observed that a history of paternal TCDD exposure reduced pulmonary alveolar space in offspring, regardless of their postnatal diet; however, a paternal fish oil diet normalized the pulmonary alveolar space of these pups (Figure 3A).

We used lung MLI to confirm that postnatal formula supplementation and paternal history of TCDD exposure independently reduced alveolar space. Formula-fed CT pups sired by standard diet fathers displayed a significant decrease in lung MLI; this trend persisted in maternal milk- and formula-fed F2TCDD pups sired by standard diet fathers (Figure 3B). CT pups did not exhibit significant changes in radial alveolar count. However, a paternal history of TCDD exposure led to a significant reduction in the radial alveolar count, which was normalized in pups whose father received a supplemental fish oil diet preconception (Figure 3C). These results confirm that historical exposure to pollution negatively impacts lung development, however, components of fish oil may mitigate this effect [65,66].

Using a novel lung injury score system, we confirmed our previous report that a history of paternal TCDD exposure increased the offspring’s risk of new BPD and that formula supplementation increased disease severity (Figure 4). These data support our hypothesis that a paternal fish oil diet preconception can reduce the risk of new BPD in offspring with a history of ancestral TCDD exposure. Our results also show that postnatal formula supplementation is associated with poor lung health outcomes [67] and a non-significant increase in the incidence of new BPD in CT mice (Figure 4A). Overall, our results support the theory that formula supplementation and paternal history of toxicant exposure are independent risk factors for the development and severity of new BPD (Table 1).

Studies have suggested that the pathophysiology of new BPD involves EMT. Therefore, to explore the potential mechanisms associated with the protective effects of fish oil against the development of new BPD, we examined E-cadherin and beta-catenin expression. These small molecules are influenced by TCDD and components of fish oil [32,33] and have each been shown to be involved in EMT. Additionally, aberrant expression of beta-catenin has been linked to new BPD [36,68,69]. Therefore, we investigated the efficacy of a paternal fish oil diet preconception in attenuating the development of new BPD by modulating the small molecules involved in EMT. We confirmed that maternal milk- and formula-fed pups with a history of paternal TCDD exposure exhibited an increased gene expression of beta-catenin (Figure 5A). We found that formula supplementation increased the gene expression of E-cadherin, independent of a paternal history of TCDD exposure. However, a paternal fish oil diet insignificantly reduced the gene expression of E-cadherin in offspring with a history of TCDD exposure (Figure 5B). Pups with a history of TCDD exposure displayed a significant increase in the co-expression of beta-catenin and E-cadherin at the gene level—a potential marker for EMT [70,71]. However, beta-catenin and E-cadherin protein expression were aberrant between groups (Figure 5D,E). Our results suggest that new BPD in offspring with a history of TCDD exposure may be associated with increased beta-catenin gene expression, supporting previous findings by others that this small molecule is dysregulated in new BPD. Surprisingly, our results also suggest that the protein expression of beta-catenin and E-cadherin may play a less significant role in the attenuation of new BPD.

Overall, our study suggests that a preconception fish oil diet in males, following in utero TCDD exposure, reduces the offspring’s risk of developing new BPD and that this effect is mediated in part through the modulation of beta-catenin gene expression, a small molecule involved in EMT. Although E-cadherin is also involved in EMT, its aberrant expression between groups suggests that it does not play a major role in new BPD outcomes. We theorize that a paternal fish oil diet preconception reduces the risk of new BPD in offspring with a history of TCDD exposure by improving placental function, which eliminates the risk of delayed postnatal growth in offspring and subsequently improves lung development. It is also likely that a paternal fish oil diet increases the levels of fish oil components (e.g., Docosahexaenoic acid (DHA) and Eicosapentaenoic acid (EPA)) in seminal fluid [72], which may, in turn, contribute to the fatty acids present in the intrauterine environment. DHA and EPA are critical to infant health and the development of lungs and other organs; however, PUFA stores are often depleted after birth following changes in the nutritional content of an infant’s postnatal diet [73,74]. Relevant to public health, this study suggests that a paternal fish oil diet may be an efficacious preventative measure in attenuating the risk of new BPD in the offspring of fathers who have been exposed to toxicants via smoking or as a consequence of their occupation.

## Figures and Tables

**Table 1:** Incidence of pups with BPD across all groups. CT, control; FO, fish oil.

Exposure Group	Incidence of New BPD	Average Lung Injury Score
CT	0/7 = 0%	1
CT + FO	0/7 = 0%	1
CT + FORMULA	1/16 = 16%	3
CT + FO + FORMULA	1/6 = 16%	2
F2TCDD	6/7 = 85%	6
F2TCDD + FO	2/8 = 25%	3
F2TCDD + FORMULA	5/7 = 71%	7
F2TCDD + FO + FORMULA	1/10 = 10%	3

## Data Availability

All relevant data is within the manuscript.

## Supplementary Materials

The following are available online at https://www.mdpi.com/article/10.3390/toxics10010007/s1: Figure S1, Full immunoblot. Table S1, *p*-values representing differences in growth curve measurements between groups. CT, Control; FO, Fish oil.
