# Supplementary material for: A Preconception Paternal Fish Oil Diet Prevents Toxicant-Driven New Bronchopulmonary Dysplasia in Neonatal Mice
Source: Toxics. 2021 Dec 27;10(1):7. doi: 10.3390/toxics10010007 (PMC8778469; doi:10.3390/toxics10010007)
Supplement: Supplementary file 1 [file toxics-10-00007-s001.zip › toxics-1439332-SM-1.20.pdf]

toxics

Supplementary Materials: A Preconception Paternal Fish Oil Diet Prevents Toxicant-Driven New Bronchopulmonary Dysplasia in Neonatal Mice

Jelonia T. Rumph, Kayla J. Rayford, Victoria R. Stephens, Sharareh Ameli, Pius N. Nde, Kevin G. Osteen and Kaylon L. Bruner-Tran

Table S1. *p*-values representing differences in growth curve measurements between groups. CT, Control; FO, Fish oil.

Day 7	Growth Curve *p*-values
CT vs. CT Formula	>0.9999
CT vs. CT + FO	0.5393
CT vs. CT + FO + Formula	0.5393
CT vs. F2TCDD	>0.9999
CT vs. F2TCDD + FO	0.9649
CT vs. F2TCDD + Formula	0.1541
CT vs. F2TCDD + FO + Formula	0.1541
**Day 8**	
CT vs. CT Formula	>0.9999
CT vs. CT + FO	0.5393
CT vs. CT + FO + Formula	0.5393
CT vs. F2TCDD	0.9649
CT vs. F2TCDD + FO	>0.9999
CT vs. F2TCDD + Formula	0.1541
CT vs. F2TCDD + FO + Formula	0.5393
**Day 9**	
CT vs. CT Formula	>0.9999
CT vs. CT + FO	0.9649
CT vs. CT + FO + Formula	0.9649
CT vs. F2TCDD	0.9649
CT vs. F2TCDD + FO	0.5393
CT vs. F2TCDD + Formula	>0.9999

CT vs. F2TCDD + FO + Formula	0.5393
**Day 10**	
CT vs. CT + FO	0.9649
CT vs. CT + Formula	0.5393
CT vs. CT + FO + Formula	0.5393
CT vs. F2TCDD	0.1541
CT vs. F2TCDD + FO	0.5393
CT vs. F2TCDD + Formula	0.9649
CT vs. F2TCDD + FO + Formula	0.5393

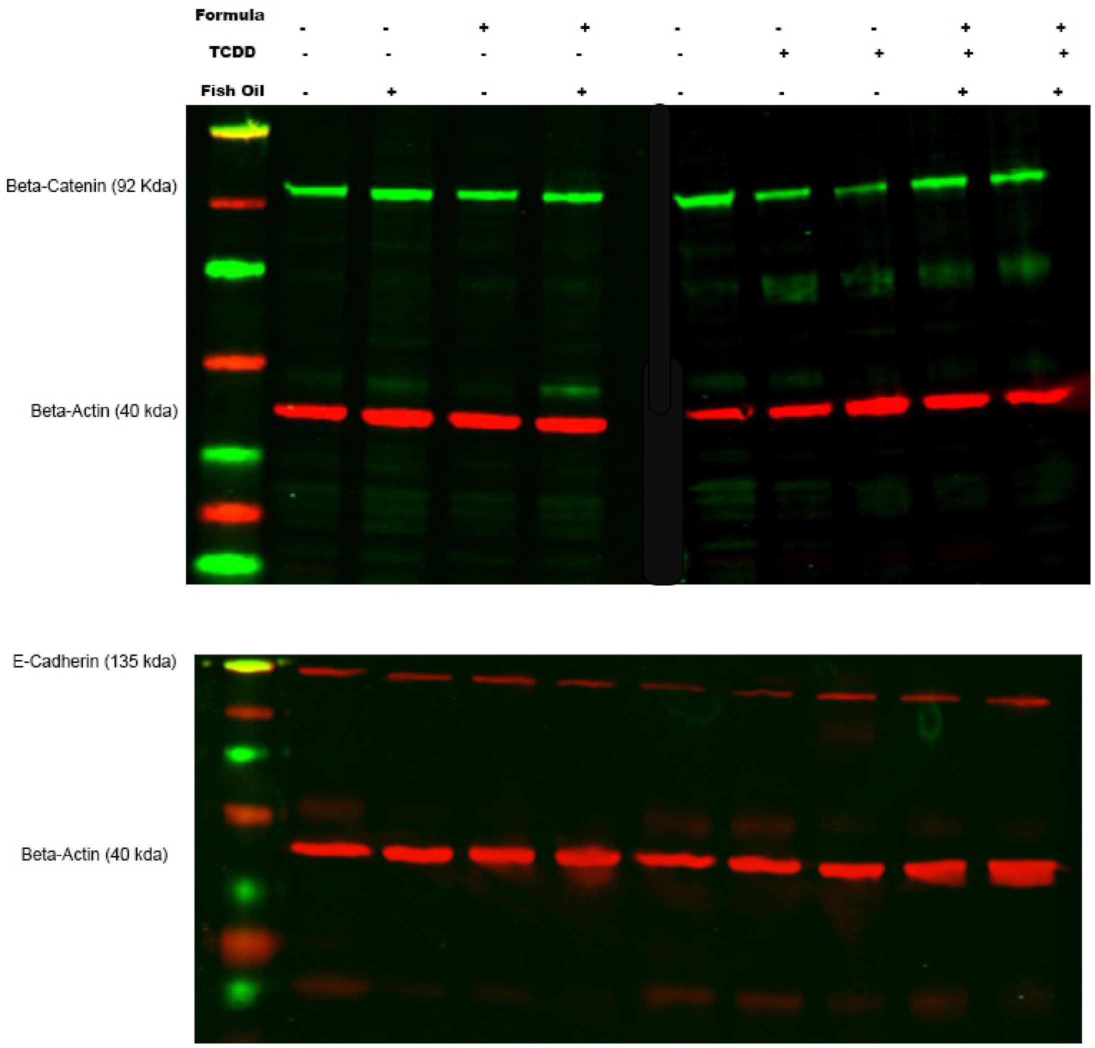

Figure S1. Full immunoblot.
